# How has the University Community Been Coping During the COVID-19 Pandemic? An Iranian Survey

**DOI:** 10.3389/fsoc.2021.645670

**Published:** 2022-01-18

**Authors:** Fereshteh Ahmadi, Önver A. Cetrez, Sharareh Akhavan, Mohammad Khodayarifard, Saeid Zandi

**Affiliations:** ^1^ Faculty of Health and Occupational Studies, University of Gävle, Gävle, Sweden; ^2^ Faculty of Theology, Uppsala University, Uppsala, Sweden; ^3^ School of Health and Welfare, Mälardalen University, Västerås, Sweden; ^4^ Faculty of Psychology and Education, University of Tehran, Tehran, Iran; ^5^ Faculty of Psychology and Education, Allameh Tabataba’i University, Tehran, Iran

**Keywords:** academic staff, academics, coping strategies, coronavirus, meaning-focused coping, meaning-making coping, religious coping, university population

## Abstract

**Objectives:** The present study, one of the first to look at COVID-19 and coping in Iran, aimed at mapping, describing and understanding the coping methods academics employ as protective resources to deal with the psychological challenges and social isolation during the COVID-19 pandemic. We specifically aimed at identifying the meaning-making coping methods used and understanding the influence of culture. The guiding research question has been: Are there differences in meaning-making coping methods by gender, age group, work/student status, and place of residence?

**Design:** The study, which used convenience sampling, was a quantitative inquiry. It employed a modified version of the RCOPE scale among faculty/staff members and students in Iran (n = 196, 75% women).

**Results:** The most frequently used coping method among all subgroups of the study sample was *thinking that life is part of a greater whole*, followed by *praying to Allah/God*. The least used coping methods were the negative religious ones. Gender differences were found for *being alone and contemplating*, stronger for men. *Thinking that life is part of a greater whole* was found mainly among on-campus students. *Praying to Allah/God* was most common among the youngest staff and students, as well as among women. Two segments of respondents were discovered—the Theists and Non-theists—where the former used more religious coping methods, were more likely to be women, older staff and students, on-campus students, married, have children, and lived in capital.

**Conclusions:** Our conclusion is that the RCOPE methods, which include religious and spiritual meaning-making methods, are of great importance to the studied Iranian informants. However, they use some secular existential meaning-making coping strategies too. This is explained by the role of religion in the larger orientation system and frame of reference in parallel with a secular worldview. Further, a sharp distinction between religious and secular worldviews was not found, which is explained by the fact that secular norms are hardly internalized in ways of thinking in Iran.

## Introduction

We live in a frightening time, in the midst of a worldwide crisis. On March 11, 2020, the World Health Organization (WHO) declared COVID-19 as a pandemic. The coronavirus has changed our world in different ways, and the changes will probably persist for many years. The psychological and psychosocial impact of the COVID-19 pandemic may be different in different parts of the world, due to lockdown strategies, duration of lock down and even the strength of countries’ regulations. Public health policies emphasizing social distancing, stay-at-home orders and individual behaviour change (e.g., hand hygiene and wearing a mask) have slowed the trajectory of COVID-19. On the other hand, the psychosocial and mental health consequences of staying at home, social distancing and social isolation may be substantial and extensive. Scientists have discussed this as a global health challenge. There is risk that the outbreak will create a “second pandemic” of mental health crises in health systems and communities (Choi et al., 2020; Ho et al., 2020; Parrish, 2020). The WHO declared: “Fear, worry, and stress are normal responses to perceived or real threats, and at times when we are faced with uncertainty or the unknown. So it is normal and understandable that people are experiencing fear in the context of the COVID-19 pandemic” (World Health Organization, 2020). The studies conducted during 2020 have shown that substance use, depression, anxiety, child abuse, domestic violence and suicide have increased during the ongoing pandemic (Galea et al., 2020). Researchers and clinicians have warned about the COVID-19 pandemic’s impact on mental health (Cao et al., 2020; Gruber et al., 2020; Marroquín et al., 2020; Pfefferbaum and North, 2020; Van Bavel et al., 2020). The fear, anxiety and anger associated with unemployment and decreased income are among the many mental health issues related to COVID-19 crisis. For people who have previously experienced trauma, posttraumatic stress disorder (PTSD) will escalate. Symptoms such as fear of germs and increased hand washing will increase among people with obsessive compulsive disorder. Feelings of fear, sadness, isolation, anxiety and financial difficulties may lead to depression and increased use of alcohol and illicit drugs (Parrish, 2020). The psychosocial impacts of the disease, together with forced quarantine in the form of nationwide lockdowns to fight against COVID-19 epidemic, may in the long run lead to acute panic, anxiety, obsessive behaviours, hoarding, paranoia, depression, and PTSD (Dubey et al., 2020). During the pandemic, negative emotions and sensitivity to social risks have increased, while scores for positive emotions and life satisfaction have decreased (Li et al., 2020). A Chinese study with 1,060 participants showed that more than 70% of respondents have moderate and higher levels of psychological symptoms, specifically elevated scores for obsessive compulsion, interpersonal sensitivity, phobic anxiety, and psychoticism (Tian et al., 2020). Some studies have studied the coping strategies people use to overcome the challenges caused by COVID-19 (e.g., Dawson and Golijani-Moghaddam, 2020; Ahmadi et al., 2021a; Ahmadi et al., 2021b; Götmann and Bechtoldt, 2021). The findings of these studies suggest that coping methods may help individuals be more prepared and empowered in facing the worries caused by the COVID-19 pandemic.

At this point, most of the studies about coping with coronavirus pandemic have been conducted in Western cultures. To date there are very few corresponding studies in Middle Eastern countries among Islamic cultures, such as the Shia Muslims of Iran (for more information about the sociocultural characteristics of the Iranian society, see the section “Coping and culture” below). Iran reported its first confirmed cases of COVID-19 infection in Qom on February 19, 2020. In response to the pandemic, the government cancelled public events and closed shopping centres, schools, universities, and holy shrines. The National Headquarters for Fighting Coronavirus ordered academic organizations to launch online educational systems. So far, university classes and exams have been held on online platforms. The government later declared a ban on travel between cities following a rise in the official case counts (Ministry of Health and Medical Education, 2020a). Iran was among the top three countries at the beginning of the pandemic and is still among the countries with high cases and deaths (Worldometers, 2020). The official number of cases has surpassed 815,117, with over 43,417 deaths recorded by November 20, 2020 (Ministry of Health and Medical Education, 2020b; Worldometers, 2020).

The Office of Social and Cultural Studies of Tehran Municipality (2020) conducted a survey among 1,023 Iranians, asking about their conditions during the coronavirus pandemic. The findings of the study showed that 51% of respondents reported serious anxiety; 20% reported suffering from moderate anxiety; and 29% reported low anxiety. The results also demonstrated that the families’ economical resilience is at a low level; 70% of participants reported having financial problems. This research also demonstrated that, in 16% of the families, family tensions have increased due to staying at home. In 58% of the families, the tension between couples has increased. Also, 46% of the families have experienced increased tension between parents and children. In another study, Farahati (2020) argued that staying in a small space reduces people’s tolerance, and statistics show an increase in divorces in Iran during the coronavirus epidemic. Mirzaei and Rahmani (2020) argued that, because Iran has a strong family orientation, the perception of home is not limited to a physical place, and staying at home means gathering all members of the extended family in a safe place. Thus, this perception will probably increase the number of trips and the traffic instead of decreasing them. In other words, Iranians may turn to visiting their parents and grandparents as a way of coping with their anxiety about conditions during the pandemic. Findings from another Iranian study conducted by the Research Center for Culture, Art and Communications (2020) showed that 19% of respondents listened to music during quarantine to relax the tensions caused by the pandemic. In addition, 18% of respondents used reading books as a way to reduce anxiety and stress, and 19% said they have gained peace of mind by talking to family members. Additionally, Alizadehfard and Saffarinia (2020) investigated the predictability of mental health based on anxiety and social cohesion arising from the coronavirus epidemic. Their results revealed that as feelings of social cohesion increase, mental health improves. Perceived national empathy and social cohesion help people to better cope with the current situation.

As mentioned, studies reveal a variety of coping mechanisms Iranian people use in facing the worries caused by the COVID-19 pandemic. However, little research attention has been paid to the use of *meaning-making coping methods* during the COVID-19 pandemic. Meaning-making coping, as explained below in the theoretical framework, refers to coping methods related to existential questions, encompassing the whole range of religious, spiritual, and secular existential coping methods. To address this gap, this study identifies the meaning-making coping methods used by Iranian academics when working and studying from home during the current crisis. By academics, we mean all individuals working or studying in academic settings (i.e., faculty/staff members and university students). It should be mentioned that we are aware that there are differences between university students and university faculty/staff members in terms of, for example, age, status, as well as values, attitudes, etc. But, they have two characteristics in common, which we were interested in: 1) they were highly educated and 2) they faced the same situation i.e., COVID-19-related challenges and teleworking (distance teaching and learning) in the framework of the university tasks, which had brought about certain socio-psychological problems for them. The aim of this study is to map, describe and understand the coping methods the university community in Iran “brings with them” as protective resources to the psychological challenges of COVID-19 and the potential negative effects of social isolation. The specific research question guiding our study is: Are there differences in meaning-making coping methods by gender, age group, work/student status, and place of residence?

## Theoretical Framework

### Coping and Culture

Generally, coping can be defined as a process of managing the discrepancy between the demands of the situation and one’s available resources—a process that can alter the stressful problem or regulate the emotional response. Lazarus and Launier (1978) defined coping as the efforts, both action-oriented and intrapsychic, a person makes to manage (i.e., master, tolerate, reduce, minimize) environmental and internal demands, and the conflicts between them, that tax or exceed his/her resources. Coping may also be defined as the process through which individuals try to understand and deal with significant demands in their lives (Ganzevoort, 1998, p.260) or as a search for significance in times of stress (Pargament, 1997, p.90). According to Hobfoll (1988, p.16), coping constitutes behaviours employed to reduce strain in the face of stressors. There are various resources individuals may bring to coping, such as material resources (e.g., money), physical resources (e.g., vitality), psychological resources (e.g., competence), social resources (e.g., interpersonal skills) or spiritual (e.g., feeling close to God) (Pargament, 1997) and existential (e.g., feeling one with nature) resources (Ahmadi, 2015).

The resources people bring with them to a coping situation help to form their orienting system, which may be defined as a general way of understanding and dealing with the world around them, which consists of relationships, habits, beliefs, values and personality (Pargament, 1997). An orienting system represents the way in which culture impacts on the individual’s life and, therefore, the way in which she/he copes with stress when facing a stressful situation, like the one caused by a pandemic. It is therefore the sociocultural context that affects the coping process. The way in which different social groups respond to stress and negative experiences is surely affected by their social background and cultural elements of the community they live in.

According to the importance of culture in the way people deal with crises and that the present study concerns an Iranian community, an overview of the sociocultural characteristics of Iranian society is noteworthy. Iran (also called Persia) is a multi-cultural, multi-ethnic, multi-language and multireligious country. The majority of the population speak Persian (official language of the country); however, the Azerbaijani language (Azeri Turkish), Kurdish, Luri, Balochi, and Mazanderani are also spoken informally. Islam is the dominant religion. Ninety eight percent of Iranians are adherents of Islam (Shi’a 89%, Sunni 9%); Iranian Sunni citizens are primarily concentrated in the provinces of Golestān, Kurdistan, and Baluchestan (Ahmadi et al., 2018). The remaining 2% are non-Muslims, such as Christians, Zoroastrians, Jews, Baháʼís, Mandeans, and Yarsanis. Shia Islam is the official religion of the country. Concerning the ethnic groups, according to Federal Research DivisionLibrary of Congress, 2008, Iran is culturally a diverse society. The predominant ethnic and cultural group consists of native speakers of Persian who are of mixed ancestry. Besides, the country has important Turkic and Arab elements in addition to the Kurds, Baloch, Bakhtyārī, Lurs, and other smaller minorities (Armenians, Assyrians, Jews, Brahuis, and others).

### Meaning-Making Coping

**FIGURE 1:**
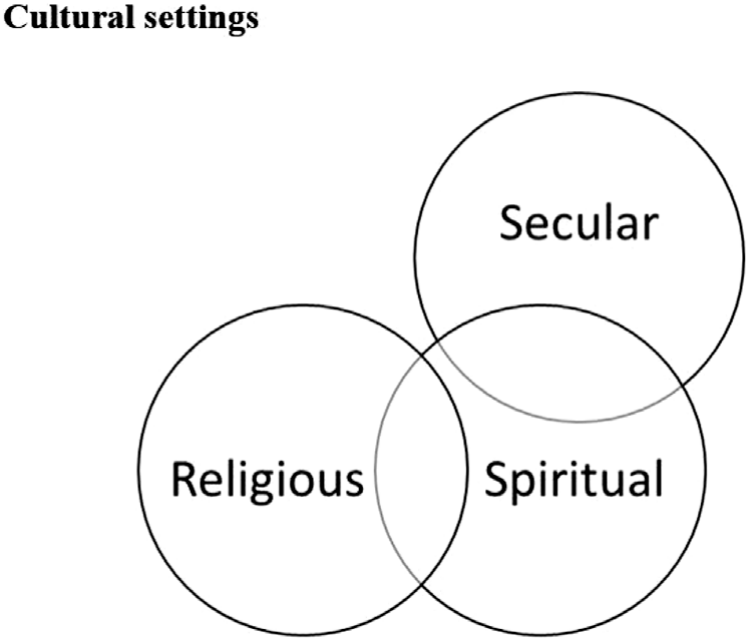
Relation of meaning-making domains (Ahmadi and Ahmadi, 2018, p.136, p.136).

According to Folkman (2008), meaning-focused coping is in its essence, an appraisal-based coping in which the person draws on his or her beliefs (e.g., religious or spiritual), values (e.g., “mattering”), and existential goals (e.g., purpose in life) to motivate and sustain coping and well-being during a difficult time. The existential questions play an important role here. Meaning making process encompasses altering one’s own perceptions by reappraising the situation and in this process, some religious/spiritual explanations are found for why stressful life event(s) has occurred or is occurring. Pargament et al. (2000) developed RCOPE instrument, a well-known measure of religious coping with major life stressors, to measure the religious/spiritual coping mechanisms. Researchers usually use the terms “religious” and “spiritual” to address meaning-focused coping methods that are based on existential issues. However, the results of several studies (Ahmadi, 2006; Ahmadi and Ahmadi, 2015; Ahmadi et al., 2016; Ahmadi and Ahmadi, 2018; Ahmadi et al., 2018) have shown that, when facing a crisis, people use other meaning-focused coping strategies that cannot be regarded as religious or spiritual, for instance, strategies connected to nature. These coping methods are defined as secular existential in nature, referring to a search for meaning. Here meaning has no connection either to religion or religious symbols, or any obvious connection to a sacred religious/spiritual source. Because these methods of coping with a crisis concern individuals’ efforts to find an inwardly source—in nature, in themselves or in others—the term secular existential coping is used to categorize them. Ahmadi and Ahmadi (2018) use the term meaning-making coping to refer to the entire spectrum of religious, spiritual, and secular existential coping methods. As they pointed out, crises typically cause an existential vacuum that requires elaboration of the old order into a new order—a new order that can help people fill this vacuum. One important point here is how, from this perspective, these three kinds of meaning-making coping strategies are connected to each other. Figure 1, presented by Ahmadi and Ahmadi (2018), shows the relation between religious, spiritual and secular existential coping orientations as they are understood in our study.

According to this model, the concepts and topics belonging to the religious and spiritual domains are somewhat overlapping. The concepts and topics of spiritual and secular existential meaning-making coping overlap, but note that there is no overlap between the secular existential and religious concepts and topics (Ahmadi and Ahmadi, 2018, p.136). This is the case because religion is defined as *a search for significance that unfolds within a traditional sacred context* (Ahmadi, 2006, p.72) and spirituality as *a search for connectedness with a sacred source that is related or not related to God or any religious holy sources* (Ahmadi, 2006, p.72–73)*.* As Ahmadi and Ahmadi (2018) pointed out, *secular meaning-making coping hardly has any point of connection with a traditional sacred context, but can overlap with a search for connectedness with a sacred source without relating to God or any traditional religious context. As mentioned before, sacred here is not defined in a religious context, but an inwardly sanctification context.* (p.34)

**FIGURE 2:**
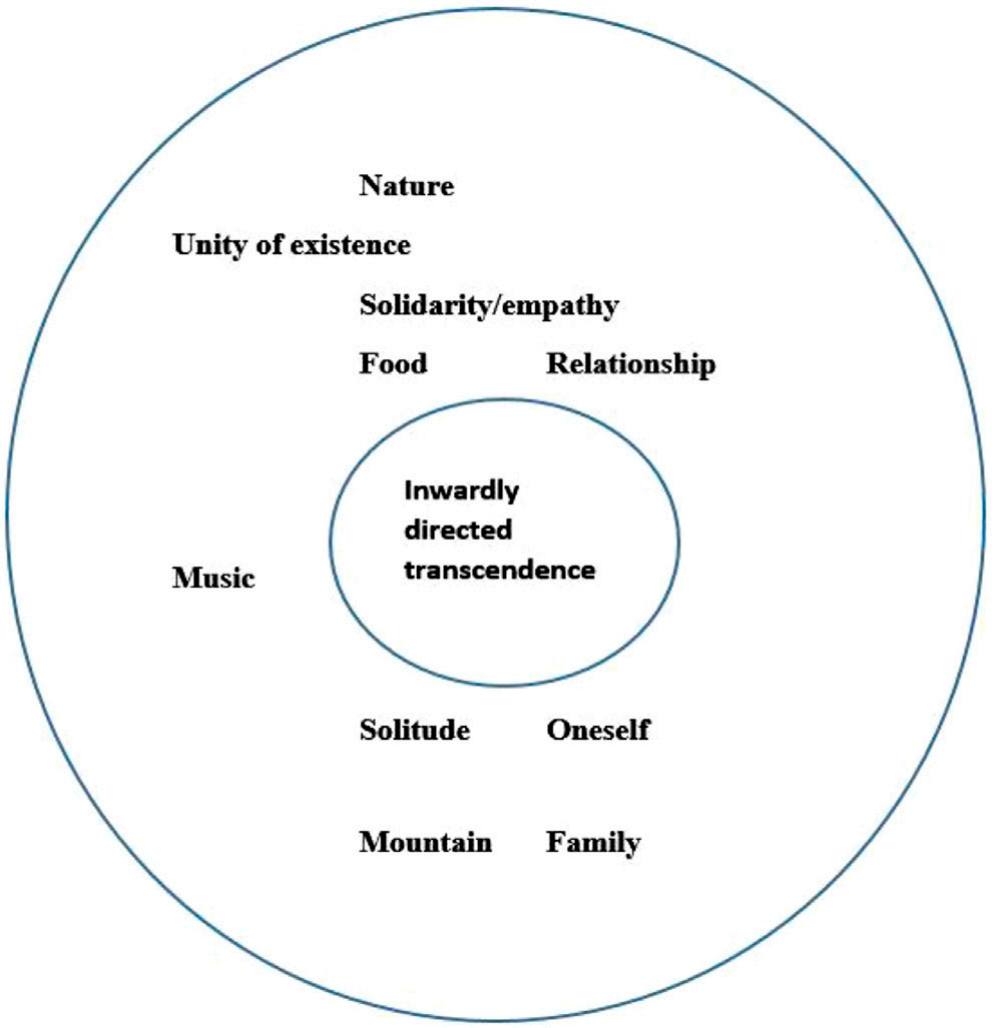
Sacred core and ring (Ahmadi and Ahmadi, 2018, p.130, p.130).

Ahmadi and Ahmadi (2018, p.130) made a distinction between theistic sacred objects and nontheistic sacred objects. Indeed, in contrast to the set of rings presented by Pargament et al. (2017, p.2), which has the outwardly transcendent as its sacred core (theistic sanctification), Ahmadi and Ahmadi presented a different set of “sacred rings” (see Figure 2). In this model, inwardly transcendence shapes the sacred core (nontheistic sanctification). In our study, we have applied this view and therefore used the term meaning-making coping to refer to the entire range of religious, spiritual and secular existential coping methods.

## Methods

### The Present Study

A quantitative research design was employed to conduct this cross-sectional study. The variables included meaning-making coping methods, gender, age group, work/student status, and place of residence.

### Sample Characteristics

The target group for this study was academics (faculty/staff members and college students) studying/working at different universities in Iran. A list-based sampling frame was chosen for this study. This approach was found most useful as the academic groups were homogenous and for which e-mail addresses were available (Fricker, 2016). The sample size was 196. Table 1 demonstrates the demographic characteristics of the participants. In this table, different age groups have been used; the first 1) is evenly distributed, but leans more towards the younger ages, while the other 2) reflects young, middle and older ages, but is not evenly distributed. A clear majority of respondents are women and almost half younger than 30, another half under 60 years, and very few are older. As expected, the clear majority has a university education and was born in and resides in Iran. While 2 in 5 respondents are distance-learning students, another 1 in 5 study on campus. One in 4 is employed full-time and 1 in 7 part-time. While the large majority, almost 2 in 3, is single, more than 1 in 3 are married, and very few are divorced or engaged. A clear majority, more than 3 in 4, does not have any children. Finally, almost 2 in 3 live in the capital, followed by almost 1 in 5 living in a medium-large and small town, but close to a large city, and a very few living in a small town, far from a large city.

**TABLE 1:** Sample characteristics (n = 196), by percentage.

Variable	Variable value	
Gender	Male	25
	Female	75
Age groups 1	<25 years old	31
	25–35 years old	39
	>35 years old	30
Age groups 2	<30 years old	47
	30–59 years old	50
	>59 years old	3
Education	High school or similar	2
	University	98
Country of birth	Iran	99
	Afghanistan	1
Country of residence	Iran	99
	Switzerland	1
Work/student status	Full-time employment	25
	Part-time employment	14
	Campus student	22
	Distance learning student	39
Civil status	Married	38
	Divorced	2
	Engaged	4
	Single	57
Children	Children	23
	No children	77
Place of residence	Capital	58
	Medium–large city	17
	Small town, close to a large city	19
	Small town, far from a large city	5

### Data

The data were collected through an Iranian online survey maker (www.cafepardazesh.com). The link to the online survey was e-mailed to potential participants. In the email, there was an invitation letter explaining the research project and asking the addressees to voluntarily participate in the project. As mentioned before, we recruited our participants from an existing list. The list included 885 academics (faculty/staff members and college students) and we sent the invitation email to all people on the list on May 30, 2020. A total of 210 female and male individuals returned the questionnaire until June 9, 2020, when the data gathering was terminated. Due to the missing data, 196 questionnaires were included in the analysis.

### Measures

The questionnaire used in this study (see Supplementary Appendix S1) was partly based on a modified version of RCOPE and partly based on the results obtained from our other studies (quantitative and qualitative) for inquiring into the applied meaning-making coping methods. The modified questionnaire encompass the entire spectrum of religious, spiritual, and secular existential coping methods. The original RCOPE, covering meaning, control, comfort/spirituality, intimacy/spirituality, and life transformation, was developed to measure manifestations of religious coping and later developed into a Brief RCOPE (Pargament et al., 2000). The instrument is multi-modal, assessing how people employ religious and spiritual coping methods. It is also multi-valent, assuming that religious coping strategies can be both adaptive and maladaptive (Pargament et al., 2011). A meta-analysis of the Brief RCOPE shows it has been mainly conducted in the United States and Western Europe using Christian samples (Pargament et al., 2011). The modified RCOPE used in the present study had a Cronbach’s Alpha value of 0.794 (high), included 15 items, and it was rated on a 4-point Likert scale ranging from 0 (“Never”) to 3 (“Very often”); an additional 10 background items were also added to the questionnaire. The instrument was validated for form, language, and content in earlier studies (Ahmadi et al., 2018; Ahmadi et al., 2021a; Ahmadi and Zandi, 2021). These studies also reported a very high reliability coefficient for this questionnaire. For the current study, the questionnaire items were modified by an Iranian expert panel to ensure that they were culturally adapted to an Iranian-Islamic context. As a result, for instance, the terms like mosque, religious leader, and Allah complemented the terms such as church, priest, and God.

### Data Analysis

Because we used convenience sampling, there are limits to the generalizability and representativeness of the results, thus no calculations of statistical significance have been conducted. We performed different calculations, including cross tabulations [by gender (female and male), age group (young, middle, older), work/student status (full-time, part-time, on-campus student, distance-learning student), and place of residence (capital, medium-large city, small town close to a large city, small town far from a large city)] and cluster analysis. The sample has not been weighted against the actual academic staff or student populations it is representative of. The data have been analysed using SPSS Statistics 27 (IBM, Chicago, IL, United States).

### Ethical Considerations

The study was conducted in accordance with the Declaration of Helsinki. In a short letter attached to the survey, the respondents were informed about the study, their ability to withdraw, and the use and preservation of the data. The respondents were also informed that responding to the survey would be regarded as giving consent. An application for ethical approval was submitted to and approved by the Swedish Ethical Review Authority for the parts of the study linked to Sweden: data analysis and data preservation (Reg. no. 2020/02,368 9). The majority of the questions had already been used in another study conducted in Iran (Meaning-making Coping among Iranians with Cancer) and been approved by the Institutional Research Ethics Committee (IR.UT. PSYEDU. REC.139.004). For the current study, an internal group at the Department of Psychology, University of Tehran (workplace of the chief investigator of the Iranian study) studied the research project and questions and approved them as well.

## Results

### Religious and Spiritual Background

**FIGURE 3:**
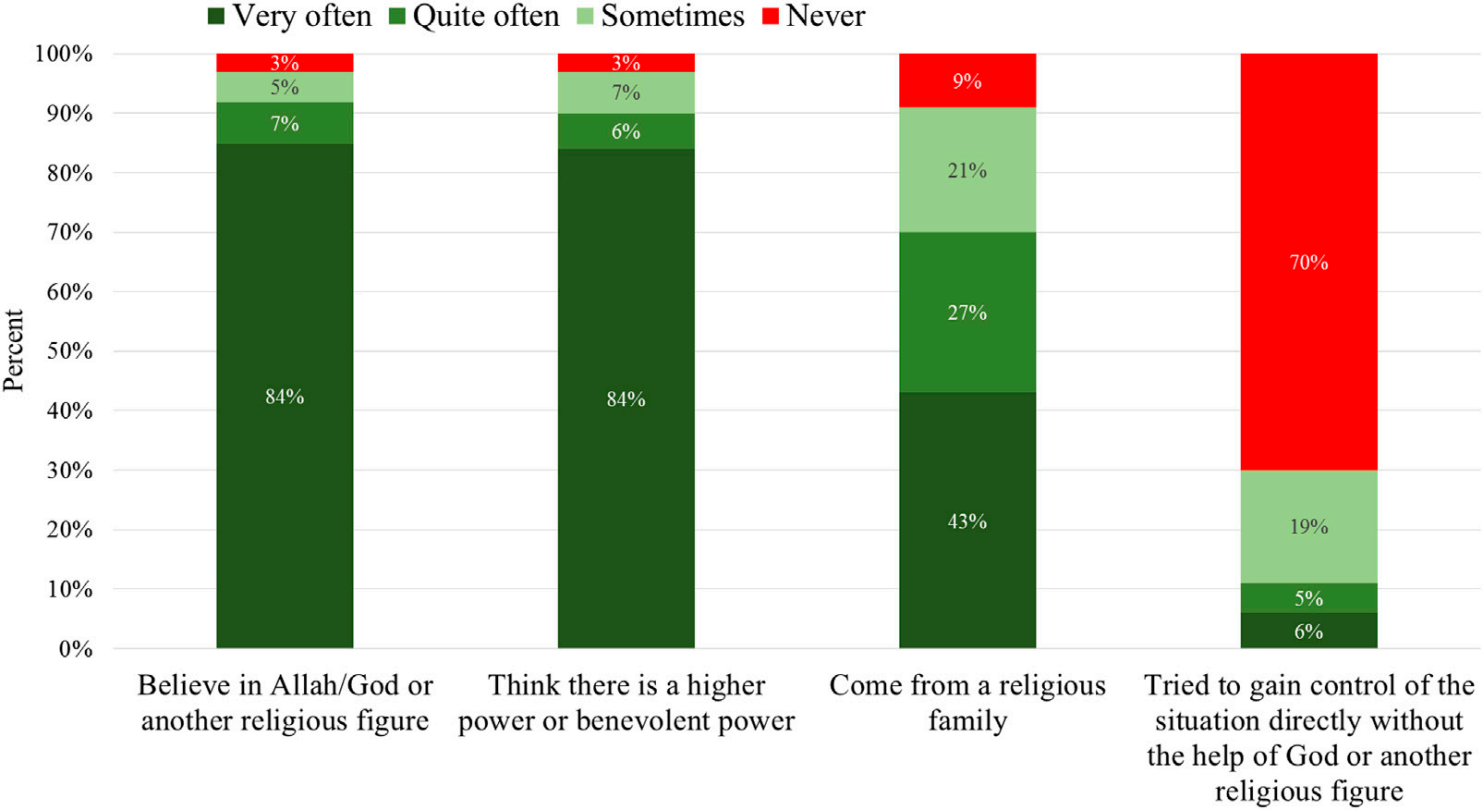
Religious and spiritual background.

Figure 3 presents the participants’ religious and spiritual background and thinking. In this academic sample from Iran, almost all claim that they believe in Allah/God or another religious figure. As many as 91% do so very or quite often, while 8% are somewhat doubting, where 5% say they only do so sometimes and 3% not at all. Figures are almost the same regarding whether they believe there is a higher power or benevolent power.

Moreover, 7 in 10 reveal that they come from a religious family, which they very or quite often admit. This means that 2 in 10 are unsure, as they also say this sometimes and 1 in 10 never. Not surprisingly, within this group of religious or spiritual people, very few try to get control of the situation without the help of Allah/God. Only 1 in 10 say that they do this often.

### Coping Methods Used Among Iranian Participants

**FIGURE 4:**
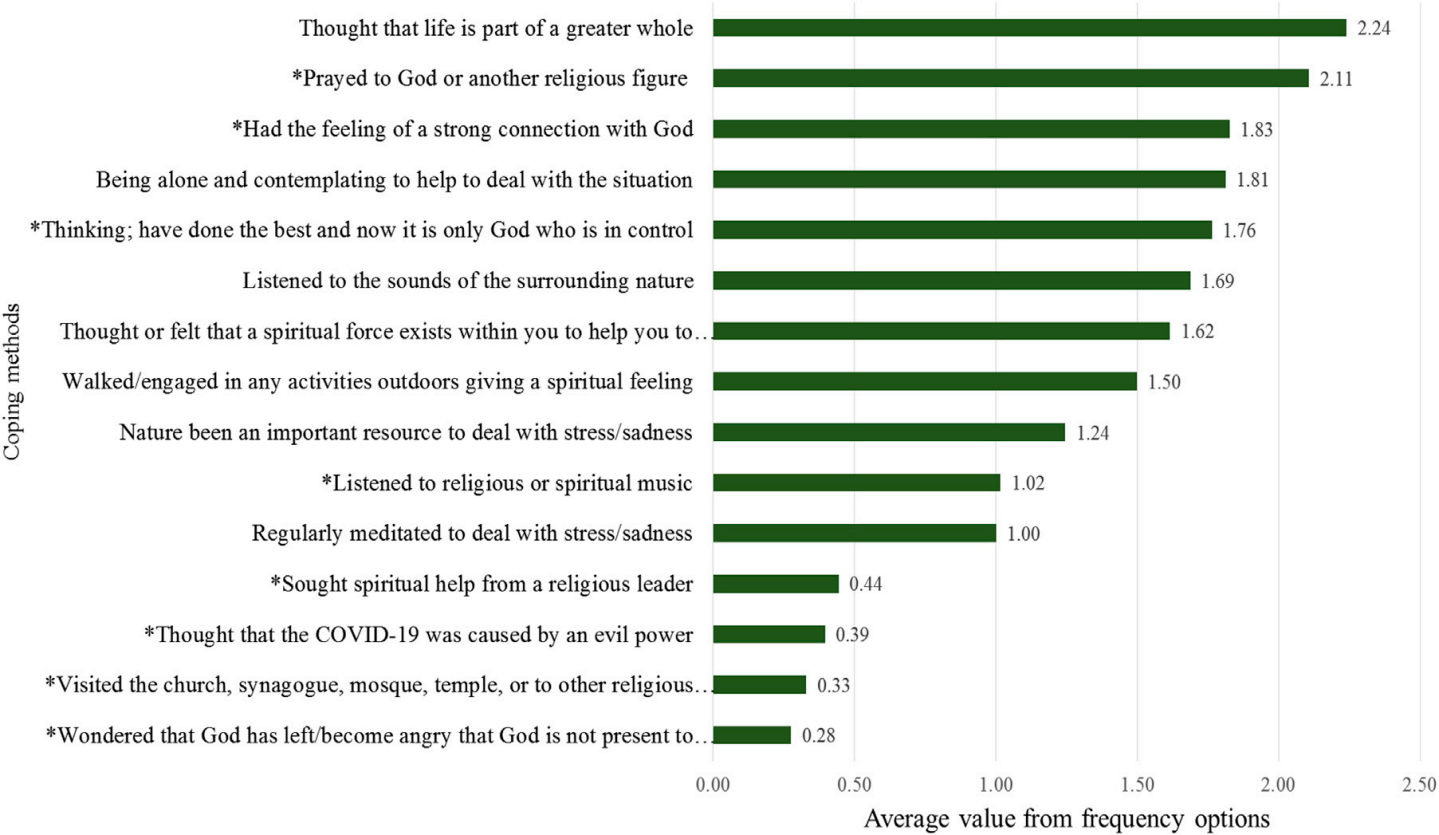
Ranking list based on frequency of meaning-making coping methods used.

Figure 4 shows the ranking of coping methods. The most common coping method for dealing with the COVID-19 crisis used in Iran among our informants is *thinking that life is part of a greater whole* (2.24 index value). This is a secular existential meaning-making coping method. The second most common method is *praying to Allah/God or another religious figure* (2.11), which is a religious meaning-making coping method. Note that index value has been based on four frequency options (never, sometimes, often, very often) regarding use of different coping methods.

Coping methods can be divided into religious and non-religious. All religious coping methods are marked with an (*) in Figure 4. Among these 15 coping methods, eight are identified as religious coping methods. These religious coping methods rank second, third, fifth, 10th, 12th-15th, which means there are as many as three among the top five and four among the top 10.

**FIGURE 5:**
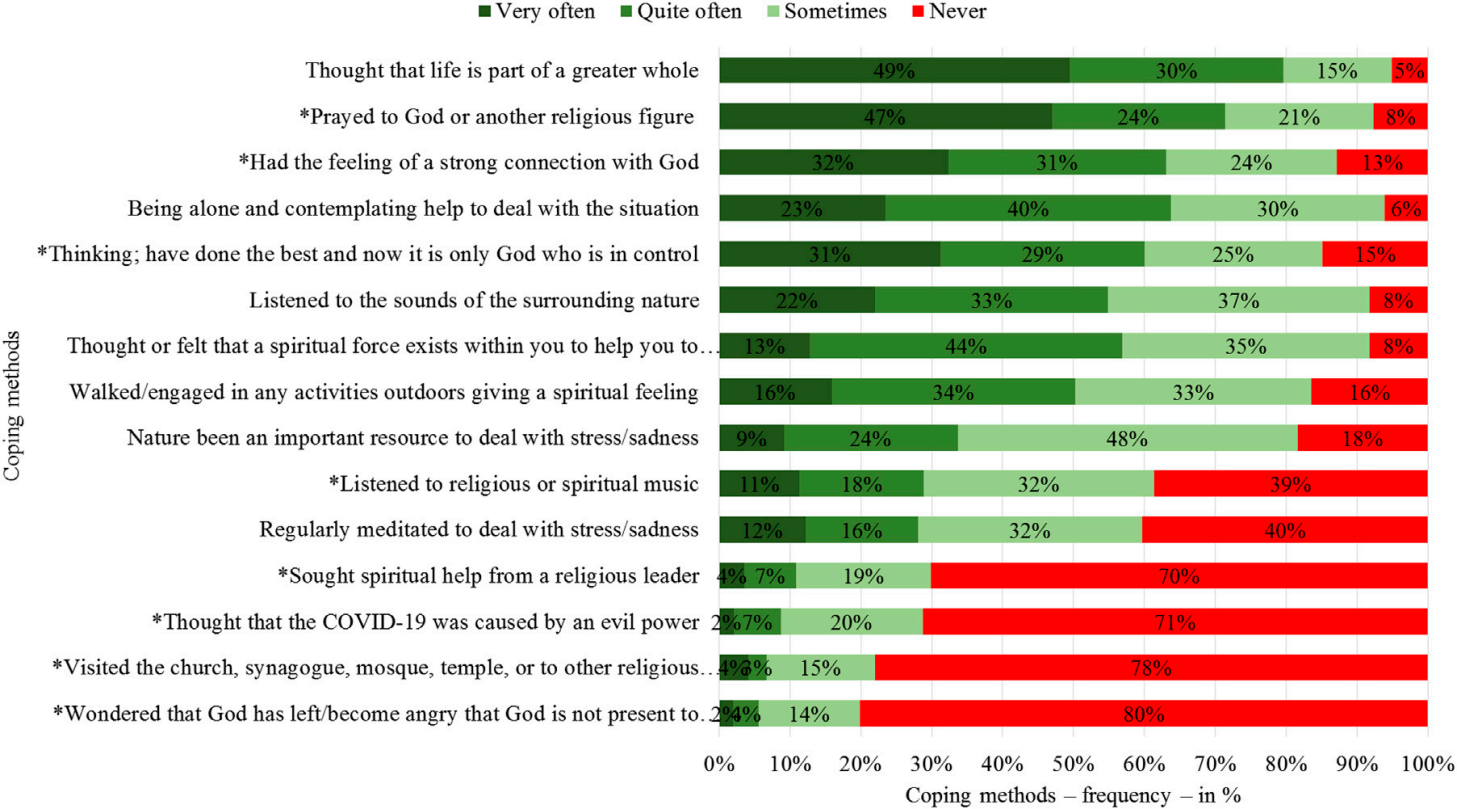
Meaning-making coping methods, by percentage.

Although an average is a good indicator and good way to rank different methods, it is important to understand how often the different techniques are used. In Figure 5, actual frequencies of use of the coping methods are shown, with the same ranking as Figure 4 based on average. Almost half of the respondents claim they have very often thought that their life is part of a greater whole or pray to Allah/God or another religious figure. Five methods are used by as many as 9 in 10 respondents.

It is also interesting that the top eight methods are used very, or at least quite, often by at least half of this group consisting of university faculty/staff members and students. The bottom four coping methods, which are religious methods, are used significantly less often than the others. More than 7 in 10 never used any of these methods.

### Coping Methods by Subgroups

Table 2 and Table 3 show the frequency average broken down by different background variables, e.g., gender, age group, work/student status and place of residence.

**TABLE 2:** Horizontal analysis comparing meaning-making coping methods, by subgroups.

		Thought that life is part of a greater whole	^a^Prayed to God or another religious figure	^a^Had the feeling of a strong connection with God	Being alone and contemplating	^a^Thinking, have done the best and now it is only God who is in control	Listened to the sounds of the surrounding nature	Thought or felt that a spiritual force exists within you to help	Walked/engaged in any activities outdoors giving a spiritual feeling	Nature been an important resource	^a^Listened to religious or spiritual music	Regularly meditated	^a^Sought spiritual help from a religious leader	^a^Thought that the COV-19 was caused by an evil power	^a^Visited the church, synagogue, mosque, temple, or other religious places	^a^Wondered that God has left/become angry that God is not present to help
Total	Total	2,24	2,11	1,83	1,81	1,76	1,69	1,62	1,50	1,24	1,02	1,00	0,44	0,39	0,33	0,28
gender	male	2,02	1,52	1,35	1,61	1,39	1,30	1,54	1,39	1,22	0,63	0,83	0,41	0,40	0,37	0,33
	female	2,31	2,29	1,97	1,87	1,88	1,81	1,64	1,53	1,25	1,14	1,05	0,45	0,39	0,32	0,26
Age groups	<25	2,17	2,33	1,80	1,67	1,92	1,71	1,56	1,25	1,13	1,02	1,08	0,42	0,52	0,27	0,30
	25–35	2,13	1,87	1,65	1,70	1,48	1,46	1,54	1,38	1,07	0,91	0,74	0,39	0,30	0,25	0,28
	>35	2,44	2,11	2,02	2,11	1,95	1,96	1,70	1,91	1,51	1,16	1,14	0,55	0,39	0,41	0,19
Age groups 1	<30	2,18	2,17	1,74	1,61	1,72	1,57	1,53	1,24	1,11	0,97	0,92	0,37	0,40	0,32	0,26
	30–59	2,27	1,99	1,80	2,02	1,75	1,83	1,62	1,71	1,34	1,09	1,01	0,53	0,36	0,29	0,27
	>59	2,43	2,43	2,71	1,57	2,43	1,57	2,00	2,00	1,14	0,86	1,14	0,43	0,86	0,29	0,00
Work/student status	Full-time employment	2,39	2,24	2,13	1,96	1,91	1,63	1,80	1,72	1,33	1,13	0,93	0,59	0,47	0,50	0,15
	Part-time employment	2,27	2,12	1,81	1,88	1,77	1,54	1,58	1,54	1,15	1,08	1,15	0,72	0,31	0,28	0,46
	Campus student	2,48	2,26	2,00	1,98	1,60	1,74	1,44	1,60	1,17	1,19	1,07	0,33	0,40	0,43	0,14
	Distance learning student	2,06	1,95	1,55	1,60	1,78	1,70	1,61	1,26	1,26	0,83	0,99	0,34	0,38	0,18	0,35
place of residence	Capital	2,38	2,22	1,96	1,91	1,84	1,70	1,68	1,57	1,13	1,06	1,07	0,50	0,40	0,38	0,26
	Medium-large city	2,29	2,18	1,91	1,74	1,82	2,00	1,58	1,74	1,41	1,15	0,91	0,47	0,56	0,32	0,18
	small town close to large city	1,86	1,81	1,50	1,58	1,64	1,29	1,39	1,17	1,44	0,80	0,89	0,28	0,28	0,22	0,28
	small town far from large city	1,80	1,70	1,20	1,80	1,10	1,90	1,80	1,00	1,30	0,80	0,90	0,30	0,20	0,10	0,80

Note.

1. Mean values are presented in table.

a Religious coping methods.

**TABLE 3:** Vertical analysis comparing meaning-making coping methods, by subgroups.

		Thought that life is part of a greater whole	^a^Prayed to God or another religious figure	^a^Had the feeling of a strong connection with God	Being alone and contemplating	^a^Thinking, have done the best and now it is only God who is in control	Listened to the sounds of the surrounding nature	Thought or felt that a spiritual force exists within you to help	Walked/engaged in any activities outdoors giving a spiritual feeling	Nature been an important resource	^a^Listened to religious or spiritual music	Regularly meditated	^a^Sought spiritual help from a religious leader	^a^Thought that the COV-19 was caused by an evil power	^a^Visited the church, synagogue, mosque, temple, or other religious places	^a^Wondered that God has left/become angry that God is not present to help
Total	Total	2,24	2,11	1,83	1,81	1,76	1,69	1,62	1,50	1,24	1,02	1,00	0,44	0,39	0,33	0,28
gender	male	2,02	1,52	1,35	1,61	1,39	1,30	1,54	1,39	1,22	0,63	0,83	0,41	0,40	0,37	0,33
	female	2,31	2,29	1,97	1,87	1,88	1,81	1,64	1,53	1,25	1,14	1,05	0,45	0,39	0,32	0,26
age groups	<25	2,17	2,33	1,80	1,67	1,92	1,71	1,56	1,25	1,13	1,02	1,08	0,42	0,52	0,27	0,30
	25–35	2,13	1,87	1,65	1,70	1,48	1,46	1,54	1,38	1,07	0,91	0,74	0,39	0,30	0,25	0,28
	>35	2,44	2,11	2,02	2,11	1,95	1,96	1,70	1,91	1,51	1,16	1,14	0,55	0,39	0,41	0,19
age groups 1	<30	2,18	2,17	1,74	1,61	1,72	1,57	1,53	1,24	1,11	0,97	0,92	0,37	0,40	0,32	0,26
	30–59	2,27	1,99	1,80	2,02	1,75	1,83	1,62	1,71	1,34	1,09	1,01	0,53	0,36	0,29	0,27
	>59	2,43	2,43	2,71	1,57	2,43	1,57	2,00	2,00	1,14	0,86	1,14	0,43	0,86	0,29	0,00
work/student status	Employed full-time	2,39	2,24	2,13	1,96	1,91	1,63	1,80	1,72	1,33	1,13	0,93	0,59	0,47	0,50	0,15
	Employed part-time	2,27	2,12	1,81	1,88	1,77	1,54	1,58	1,54	1,15	1,08	1,15	0,72	0,31	0,28	0,46
	Campus student	2,48	2,26	2,00	1,98	1,60	1,74	1,44	1,60	1,17	1,19	1,07	0,33	0,40	0,43	0,14
	Distance learning student	2,06	1,95	1,55	1,60	1,78	1,70	1,61	1,26	1,26	0,83	0,99	0,34	0,38	0,18	0,35
place of residence	capital	2,38	2,22	1,96	1,91	1,84	1,70	1,68	1,57	1,13	1,06	1,07	0,50	0,40	0,38	0,26
	medium-large city	2,29	2,18	1,91	1,74	1,82	2,00	1,58	1,74	1,41	1,15	0,91	0,47	0,56	0,32	0,18
	small town close to large city	1,86	1,81	1,50	1,58	1,64	1,29	1,39	1,17	1,44	0,80	0,89	0,28	0,28	0,22	0,28
	small town far from large city	1,80	1,70	1,20	1,80	1,10	1,90	1,80	1,00	1,30	0,80	0,90	0,30	0,20	0,10	0,80

Note.

1. Mean values are presented in table.

a Religious coping methods.

Table 2 depicts the ***analysis horizontally comparing methods within each subgroup***. The results show that the coping methods *thinking that life is part of a greater whole* and *praying to Allah/God* are the two most used coping methods among almost all subgroups. There are some interesting exceptions; for men, the coping method *being alone and contemplating to help to deal with the crisis situation* is the second most common; this is also true of the 30- to 59-year-old group. People living in a small town far from a large city most often *listen to the sounds of surrounding nature*, but as their second most common method they also *think about a spiritual force*, *spend time alone* as well as *think of life as part of a greater whole*. The bottom four religious coping methods are in principle the least often used methods for all of these subgroups.

Table 3 shows a ***vertical analysis indicating that each method is compared between the different subgroups***. The most common method, *thinking that life is part of a greater whole*, is found among on-campus students, but also among older age groups in the respective age group division. People living in a small town do this the least frequently. *Praying to Allah/God or another religious figure*, the second most used strategy, is most common among youngest groups, younger than 25 years of age, and much more common among women than among men.

### Coping Methods Used by Coping Method Segments

To further analyse the results, we applied a cluster analysis, which is a multivariate statistical technique assessing the similarities between units or assemblages, based on the occurrence or non-occurrence of specific artefact types or other components within them. It is a class of statistical techniques that can be applied to data that exhibit “natural” groupings. Cluster analysis sorts through the raw data and groups them into clusters. In our study, the two-step cluster analysis in SPSS has been used. The cluster variables used in this case are all coping methods. The two clusters are auto-generated by this statistical method and the cluster quality is fair.

**FIGURE 6:**
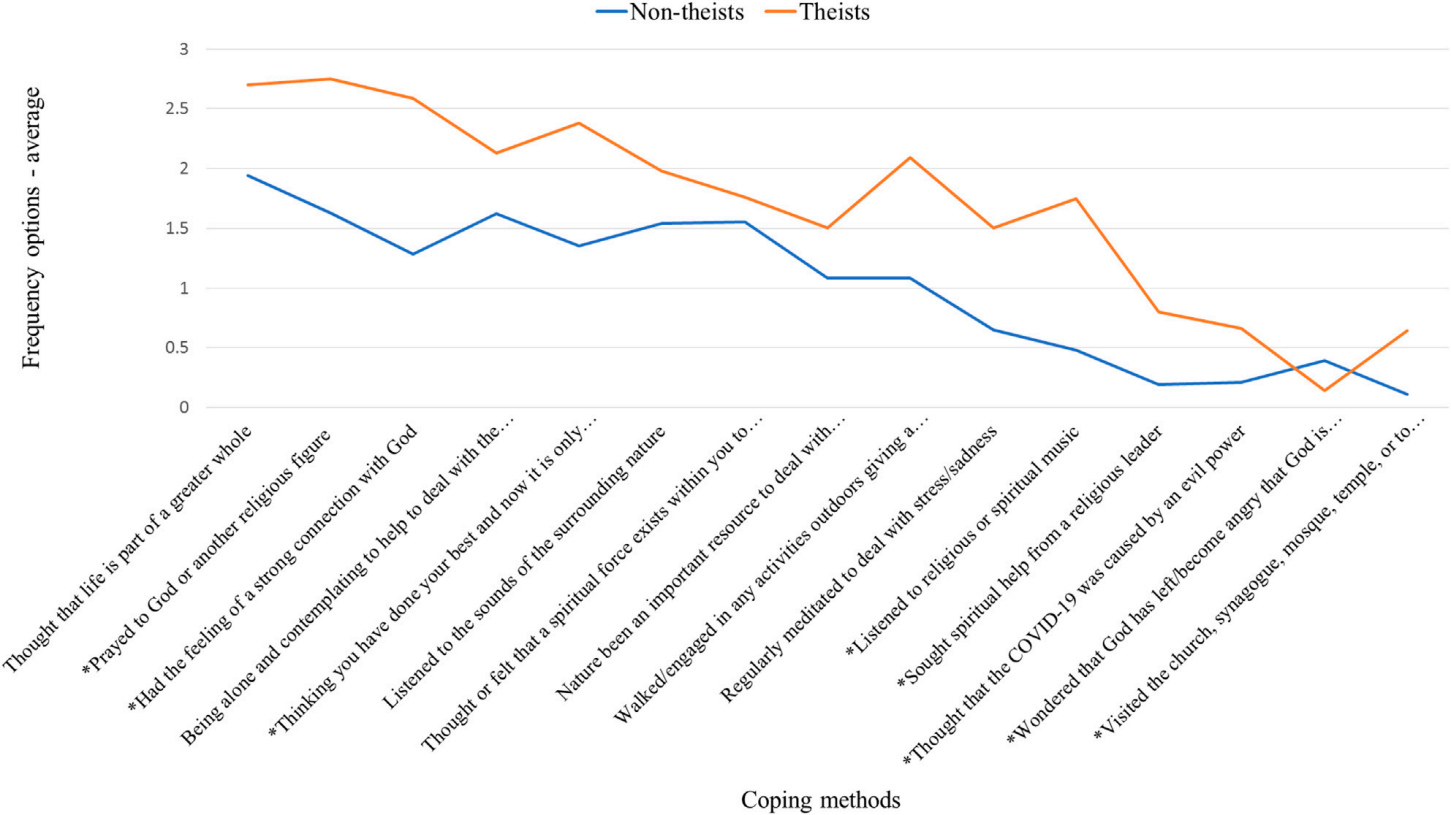
Two auto-generated segments based on frequency of use of different coping methods.

Two auto-generated segments could be found from the material. This segmentation is based on how often the different coping methods are used. Figure 6 (based on an index from 0 = Never to 3 = Very Often) shows one segment called High-frequency of coping methods, Theists, and the other segment Low-frequency of coping methods, Non-theists. Theists use all coping methods more often than the Non-theists do, except for the method thinking that *Allah/God has left them*, and *becoming angry that Allah/God is not present to help*. Both segments use the coping method thinking that life is part of something greater the most. The greatest differences between the two segments are to be found for the coping method *having a feeling of strong connection to Allah/God*. Another big difference is that Theists *listen to religious music* much more often than Non-theists do, but also *pray to Allah/God or another religious figure* more often. This means there is not only a difference in how often the two segments use different coping methods, but also that Theists use religious coping methods significantly more often.

What are the characteristics of the two auto-generated segments? The Theists are more often women, 93% compared to 65% for Non-atheists. The average age is older for Theists, as 36% are above 35 years compared to 28% among the Non-Theists, who are more often between 25 and 35 years of age. The Theists are also more often on-campus students than the Non-theists, even though both groups consist mostly of distance learning students. Both have the same shares of full- or part-time employment at the university. The older Theists are also more likely to be married, as 46% of them are married. The Non-theists are more likely to be singles (60%). The older, and more likely to be married Theists, also have children to a larger extent: 31 versus 19%. A majority of both segments live in Tehran, but Theists do so to a greater extent: 66 versus 54%. The Non-theists are more likely to live in small towns, close to or far from a large city, compared to the Theists.

## Discussion

In this study, we examined the meaning-making coping methods employed by Iranian academic workers and students to overcome the psychological difficulties caused by the COVID-19 pandemic. We should mention that the obtained results regards the informants in this study. We do not have any ambitions to generalize the results to the whole population.

Our results have shown that the most frequently used coping method is a secular existential one (*thinking that life is part of a greater whole*), followed by a religious coping method (*praying to Allah/God*); this holds true for all of the subgroups. At the same time, the negative religious coping methods are much less likely to be used than other methods. Some exceptions are found: *Being alone and contemplating* is stronger among men and older people. Additionally, our results show that *thinking that life is part of a greater whole* is found mainly among on-campus students, while *praying to Allah/God* is most common among the youngest staff and students as well as among women.

One explanation for why *thinking that life is part of a greater whole* is the most frequently used coping method among Iranian informants is the strong prevalence of the notion “the unity of existence” (*vahdat-e vojod*) in Iranian ways of thinking (Corbin, 1993; Ahmadi and Ahmadi, 1998). This idea—which is advocated by several Persian Sufis, such as Rumi who is perished and read by many Iranians—is integrated into the whole structure of the Shi’i thought of Mulla Sadra, who is one of the most important thinkers among the Shia (Corbin, 1993). As Ahmadi and Ahmadi (1998, p.69) explained, “Sufis do not believe in the nature of man as such and as separated from the nature of “Absolute”. To them, the variety in the phenomenal world is nothing but the divergent forms which the Absolute takes when He manifests His Own Self.”

As our findings show, the RCOPE methods appear to be of great importance to the Iranian informants. One possible reason for this is that religion is a larger part of their orientation system, which may be defined as a “general way of viewing and dealing with the world. It consists of habits, values, relationships, generalized beliefs, and personality. The orienting system is a frame of reference, a blueprint of oneself and the world that is used to anticipate and come to terms with life’s events. The orienting system directs us to some life events and away from others” (Pargament, 1997, pp.99–100).

We should remember that Iranian society, like most modern and dynamic societies, is characterized by a wide range of perspectives and conditions. There are many forms of religiosity and generalization is difficult. Still, some characteristics are more common than others: the importance of family, a proud commitment to local traditions, and a tendency of adhering to post-modern way of life that forms their choice of coping strategies (Ahmadi et al., 2018). This may explain the coping methods found in the present study, methods that are not religious but used frequently: *thinking that life is part of a greater whole* (79% very often and quite often, and 15% sometimes), *being alone and contemplating* (63% very often and quite often, and 30% sometimes), *listening to the sounds of nature* (55% very often and quite often, and 37% sometimes), *finding a spiritual force inside oneself* (57% very often and quite often, and 35% sometimes), *walking/engaging in any activities outdoors and finding a spiritual feeling* (50% very often and quite often, and 33% sometimes), and *nature as an important factor* (33% very often and quite often, and 48% sometimes).

As we can see, nature itself, the music of nature, and being in nature are all important secular existential meaning-making coping methods found in the present study. Nature can be used as an effective coping method in different ways (Ahmadi, 2006; Ahmadi, 2015). Here we find another feature, perhaps a more secular one: the idea of unity of existence, i.e., unity with nature.

It should also be mentioned that four of the religious coping methods were found to have the lowest frequency in the present study: *thinking that COVID-19 was caused by an evil power*; *wondering whether God has left/become angry, whether God is not present*; *visiting a church, synagogue, mosque, temple or other religious places*; and *seeking spiritual help from a religious leader*.

Of the four above-mentioned coping methods, the first two are negative religious methods. As other studies (Aflakseir and Coleman, 2011) have also shown, positive religious coping is used more frequently by Iranian informants than is negative religious coping. We should also remember that the informants belong to the highly educated strata of Iranian society, and therefore their religious beliefs tend to be more rational than superstitious, e.g., believing in the power of evil. Here we should mention that, as Ahmadi and Ahmadi (1998) mentioned, according to “Iranian Islam”, the psychological origin of evil reveals the ultimate nonexistence of evil. It is a product of human finitude. Indeed, by refusing to see evil as originating from the realm of the Divine Essence and by regarding it as a product of human finitude, the existence of any discrepancy between the imperfections of the world and the perfection of God is rejected. This being the case, it is clear that the problem of theodicy does not exist in Islam.

The two other coping methods—i.e., *seeking spiritual help from a religious leader* and *visiting a church, synagogue, mosque, temple or other religious places*—are positive religious coping methods, but nonetheless belong to those with lower frequencies. The low frequency of use of the religious coping method *seeking spiritual help from a religious leader* can be explained by referring to the absence of the phenomenon of the Church—in its Christian sense—in Islam. As Corbin (1993, p.4) mentioned, in Islam there is no clergy that possesses the “means of grace”. Islam has neither a dogmatic magisterium nor a council tasked with defining dogma. As Ahmadi and Ahmadi (1998, p.34) explained, “the religious consciousness of Islam is not concentrated on a historical fact, but rather on a meta-historical, or better, *trans*-historical fact of the primordial covenant (mithaq) between man and God as understood from the Sura 7:172 in the Qur’an”. Therefore, the clergy is not necessarily a medium between individuals and God. Another reason may be that the clergy (unlike Saints and Imams) are religious leaders who mainly give mundane advice (lead Islamic worship services, serve as community leaders, and provide religious guidance), not spiritual help.

As concerns the low frequency of use of visiting mosques, we should remember that the country was hit very hard by COVID-19, and in order to contain the coronavirus epidemic, the government closed religious sites and cancelled congregational prayers. Although visiting a mosque may not be a spiritual act, pilgrimage (Ziyarat), which refers to visiting Imams’ tombs, is (Khodayarifard et al., 2021). As Ahmadi et al. (2018) showed, it is an important form of religious coping in Iran. Ziyarat is not only an act of visiting the holy places, but also “a multi-dimensional phenomenon”, “the culture of devotion,” related to the concept of *imamat,* which is important to the Shiits (Khosronejad, 2012, p.13); but during the ongoing pandemic, the government has closed such places as well.

## Conclusion

The present survey study among academics in Iran, concerning their meaning-making coping with COVID-19, demonstrates that religious coping methods—*praying to God or another religious figure*; *having a feeling of a strong connection with God*; and *thinking I have done my best and now it is only God who is in control*—are the most prevalent methods among the informants. Regarding secular meaning-making coping, the results indicate that most common methods used by our informants were *thinking that life is part of a greater whole*, *being alone and contemplating to help to deal with the situation*, as well as *listening to the sounds of surrounding nature*.

We conclude that Iran can be a good example of how religion can provide an immediate means of coping among individuals for whom religion constitutes a major part of their orienting system. Although religion is part of the worldview of the majority, interestingly, a secular worldview and associated secular coping methods are used in parallel. The study indicates that the sharp distinction between religious and non-religious spirituality (see Figure 1), which we have found in studies on meaning-making coping in some secular countries (Ahmadi, 2006; Ahmadi, 2015; la Cour and Hvidt, 2010), can hardly be observed among the Iranian informants. The reason may be that secularism is not rooted in Iran, and despite the fact that there are some in the country who seem to be secular, secular norms and values are hardly internalized in their ways of thinking.

Several studies (Ahmadi et al., 2021a; Ahmadi, 2006, 2015; Ahmadi et al., 2021b; Ahmadi and Zandi, 2021; la Cour and Hvidt, 2010; la Cour et al., 2012) have shown that, in countries like Sweden or Denmark where people have internalized secular norms and values, when informants maintain that they are spiritual, this sometimes implies that they are neither religious nor atheists. For instance, studies have shown that, in some strong secular countries, when people say they believe in a higher power, they often are not referring to God. Along the same lines, in such countries believing that your life is part of a great whole is a coping method used by people who are spiritual, but not religious. However, as it seems in countries like Iran, Turkey and Malaysia, where secularism is not rooted in people’s ways of thinking (Ahmadi and Ahmadi, 2018; Ahmadi et al., 2018; Ahmadi and Rabbani, 2019; Cetrez et al., 2020), that sharp contrast between religiosity and spirituality hardly exists, and spiritual coping methods are often used by people as religious coping methods. Concerning Iran, besides the strong position of Islamic mysticism, Sufism may serve as an obstacle to the development of non-religious spirituality in this country.

### Strengths and Limitations of this Study

The strength of this study is attributable to its novelty as the study is timely and concerns a current topic. To our knowledge, this is the first study of its kind to investigate meaning-making coping in an Iranian university population in the midst of COVID-19 concerns and uncertainty. Also, the cultural context investigated may be not well recognized especially among readers from Western countries. One limitation of this study is the small sample size, which may reduce the explanatory power of the study and the generalizability of the findings to other populations and settings. Moreover, the sample skewed female. Another limitation is the study’s cross-sectional design; a longitudinal study design would be more useful in assessing the long-term maintenance of coping strategies used by academics.

### Some Directions for Future Research and Practical Implications

Based on our results, we suggest the following research and practical implications:

• Extending our knowledge of the importance of different meaning-making coping methods may help to improve and expand efforts in social work and social care to assist people struggling with the psychological effects of COVID-19, especially in different societies.
• There is a need to develop a public mental health approach that can provide both an interdisciplinary and a holistic framework. This will help us to deepen our understanding of the cultural factors, well-being and protective factors when examining the short- and long-term consequences of COVID-19.
• Although COVID-19 research has thus far been dominated by bio-medical studies, we need to employ culturally sensitive tools to respond to crises—tools that enable us to understand and explain how and why humans react the way they do, as well as to work preventively.

### Policy Recommendations

• Different coping methods, including secular ones, should be given more consideration by all practitioners in planning and in providing care for people facing a COVID-19-related crisis. There is a risk that, in some countries where religion is an integrated part of people’s lives, these planners and policymakers will give more room to religious/spiritual coping methods. We recommend concretely that planners and policymakers, in addition to providing facilities for use of religious coping methods for people suffering from COVID-19-related psychological problems, also allocate funds and facilities to the use of spiritual and secular existential coping methods.
• It is important for above-mentioned practitioners to improve their cultural competence regarding coping methods, including secular, religious, and spiritual methods, and the positive and negative effects such strategies may have in coping with COVID-19.

## Data Availability

The raw data supporting the conclusions of this article will be made available by the authors, if due acknowledgement is made to this study.
