# Supplementary material for: How has the University Community Been Coping During the COVID-19 Pandemic? An Iranian Survey
Source: Front Sociol. 2022 Jan 18;6:645670. doi: 10.3389/fsoc.2021.645670 (PMC8804233; doi:10.3389/fsoc.2021.645670)
Supplement: Supplementary file 1 [file DataSheet1.pdf]

Coping with COVID-19

Consent

1. I have been informed about the study “Coping with COVID-19” and I give consent to participate in this study:
Yes ☐0 No ☐1

2. I give consent that my answers will be handled as described in the information letter:
Yes ☐0 No ☐1

Coping

What do/did you do to handle the situation during the “COVID-19 time”, if you feel/felt stressed, sad, or depressed or have/had other reactions?

	Never – 0	Sometimes –1	Quite often –2	Very often –3
3. Have you thought that your life is part of a greater whole?	☐0	☐1	☐2	☐3
4. Have you thought or felt that a spiritual force exists in you to help you deal with the situation?	☐0	☐1	☐2	☐3
5. Has nature been an important resource for you in how to deal with your stress/sadness or other negative feelings?	☐0	☐1	☐2	☐3
6. Has being alone and having the chance to contemplate helped you deal with the situation?	☐0	☐1	☐2	☐3
7. Have you listened to the sounds of surrounding nature?	☐0	☐1	☐2	☐3
8. Have you walked or engaged in any activities outdoors that give/gave you a spiritual feeling?	☐0	☐1	☐2	☐3
9. Have you regularly meditated to deal with your stress/sadness or other negative feelings?	☐0	☐1	☐2	☐3
10. Have you sought spiritual help from a religious leader?	☐0	☐1	☐2	☐3
11. Have you thought that COVID-19 was caused by an evil power?	☐0	☐1	☐2	☐3
12. Have you wondered whether God has left you or become angry that God is not present to help you?	☐0	☐1	☐2	☐3
13. Have you had the feeling of a strong connection with God?	☐0	☐1	☐2	☐3
14. Have you visited a church, synagogue, mosque, temple, or other religious place?	☐0	☐1	☐2	☐3
15. Have you prayed to God or another religious figure to make things better?	☐0	☐1	☐2	☐3
16. Have you listened to religious or spiritual music?	☐0	☐1	☐2	☐3
17. Do/did you think that you have/had done your best and now it is only God who is in control?	☐0	☐1	☐2	☐3

	Never – 0	Sometimes –1	Quite often –2	Very often –3
18. Have you tried to gain control of the situation directly without the help of God or another religious figure?	□0	□1	□2	□3
19. Do you believe in God or another religious figure?	□0	□1	□2	□3
20. Do you think there is a higher power or benevolent power?	□0	□1	□2	□3
21. Would you say that you come from a religious family?	□0	□1	□2	□3

Background questions

28. What is your current work/student status?

1□ Employed full-time
2□ Employed part-time
3□ On-campus student
4□ Distance-learning student

29. What year were you born? …….

30. What is your gender?

1□ Male
2□ Female
3□ Neither of the above

31. What is your highest education level?

1□ Lower than elementary school
2□ Elementary School or equivalent
3□ High School or equivalent
4□ University or equivalent

32. What is your current civil status?

1□ Married
2□ Divorced
3□ Engaged
4□ Widowed
5□ Single
6□ other (please briefly specify)………………

33. Do you have children?

□ 0 Yes
□ 1 No

34. What characterizes the place you live?

□ 0 Capital
□ 1 Medium–large city, not capital

□ 2 Small town close to a large city
□ 3 Small town far from a large city

35. What is your country of birth? (Please write)................................

36. Country of residence: (Please write) ……………………………..
